# Medium-to-long-term success rate of left atrial appendage closure using endocardial sutures and postoperative anticoagulant strategies

**DOI:** 10.3389/fcvm.2026.1776185

**Published:** 2026-07-01

**Authors:** Wenzhong Heng, Hongying Song, Rong Zhang, Shibo Zhou, Maoxun Huang, Yong Wang, Huiying Wu, Tiance Wang

**Affiliations:** 1 Department of Cardiovascular Surgery, The Second Hospital of Jilin University, Changchun, Jilin, China; 2 Department of Ultrasound, The Second Hospital of Jilin University, Changchun, China; 3 Department of Cardiovascular Circulation, The Second Hospital of Jilin University, Changchun, China

**Keywords:** anticoagulant strategies, atrial fibrillation, cardiac procedures, left atrial appendage closure, valvular heart disease

## Abstract

**Introduction:**

Surgical endocardial closure of the left atrial appendage (LAA) may reduce thromboembolic risk in patients with atrial fibrillation (AF); however, evidence regarding its medium-to-long-term durability remains limited. This study evaluated the efficacy and safety of endocardial suture closure of the LAA during concomitant cardiac surgery.

**Methods:**

This retrospective study included 50 patients with valvular heart disease and AF who underwent valve surgery, the Cox-Maze IV procedure, and LAA closure using an endocardial suture technique between 2013 and 2018. Intraoperative transesophageal echocardiography (TEE) was used to assess immediate closure. Transthoracic echocardiography and TEE were performed during follow-up. Closure failure was defined as a residual LAA stump >1 cm or residual to-and-fro flow between the left atrium and LAA.

**Results:**

The mean follow-up duration was 7.16 ± 1.73 years. Intraoperative TEE showed no residual LAA stump or to-and-fro flow in any patient. At follow-up, no patient had a residual stump >1 cm, whereas residual to-and-fro flow was detected in 5 patients (10.0%), corresponding to a medium-to-long-term closure success rate of 90.0%. Four of these five patients (80.0%) developed thrombus within the residual LAA stump, and one experienced an ischemic stroke. No stroke occurred among patients with imaging-confirmed successful LAA closure.

**Discussion:**

Endocardial suture closure provided a high medium-to-long-term imaging success rate in this single-center cohort. Residual to-and-fro flow, even in the absence of a stump >1 cm, may be clinically relevant because it was frequently accompanied by thrombus formation. These findings should be interpreted as hypothesis-generating because of the limited sample size and low event rate. Postoperative anticoagulation decisions should remain individualized and guideline-informed.

## 1 Introduction

Atrial fibrillation (AF) is the most common arrhythmia and a significant source of thromboembolic events, increasing stroke risk by 2.4-fold compared to patients without AF (1). The left atrial appendage (LAA) is a primary site for thrombus formation in patients with AF, with studies showing that the LAA is the source of thrombus in >90% of the nonrheumatic AF cases among patients with stroke (2). Therefore, LAA occlusion or closure is crucial in preventing thromboembolic events.

Surgical left atrial appendage occlusion (S-LAAO) has been associated with reduced stroke risk in selected patients (3–5). The latest American College of Cardiology (ACC) guidelines recommend treating LAA during combined valve surgery (6). Various techniques for S-LAAO have been proposed, including surgical excision, stapled excision, and certified surgical closure devices (such as AtriClip [AtriCure, Inc., Mason, US]) (7, 8). Different results have been reported regarding these techniques. The traditional method of endocardial suture for S-LAAO, popularized in 2000, showed a 64% success rate according to transesophageal echocardiography (TEE) (9). However, subsequent reports have shown mixed success rates for the endocardial suture technique, ranging from 23% to 89.7% (10, 11). Due to their overall low success rate and the development of multiple S-LAAO methods, the application of endocardial suture techniques is currently limited.

This study aimed to investigate the effects of the endocardial closure technique on the LAA and summarize the experience of LAA closure failure. It further discussed the risk of future thrombosis and the appropriate maintenance of anticoagulant drugs to ensure a lower incidence of stroke.

## 2 Methods

### 2.1 Patient selection

The study adhered to the principles of the Declaration of Helsinki (revised in 2013) and was approved by the ethics board of the Second Hospital of Jilin University (No. 2024 [076]). From September 2013 to September 2018, 306 patients were preoperatively diagnosed with valvular heart diseases combined with AF using transthoracic echocardiography (TTE) and electrocardiogram (ECG). All patients underwent open-heart valvular surgery combined with AF radiofrequency ablation (Cox-Maze IV procedure) and LAA closure using the endocardial suture technique.

Strict inclusion and exclusion criteria were established for the study. Inclusion criteria: (1) AF diagnosis confirmed preoperatively by ECG; (2) valvular heart disease confirmed by color Doppler ultrasound and surgical indications (including valve stenosis, insufficiency affecting the heart's ability to pump blood, and ineffective drug treatment). Exclusion criteria: (1) patients with abnormal coagulation function, systemic infection, or malignant tumor; (2) severe liver or kidney dysfunction, or mental disorders; (3) refusal to undergo TEE; (4) contraindications to TEE (such as esophageal stenosis or diverticulum, or severe cervical dislocation).

### 2.2 Assessment of LA/LAA thrombus

**Figure 1:**
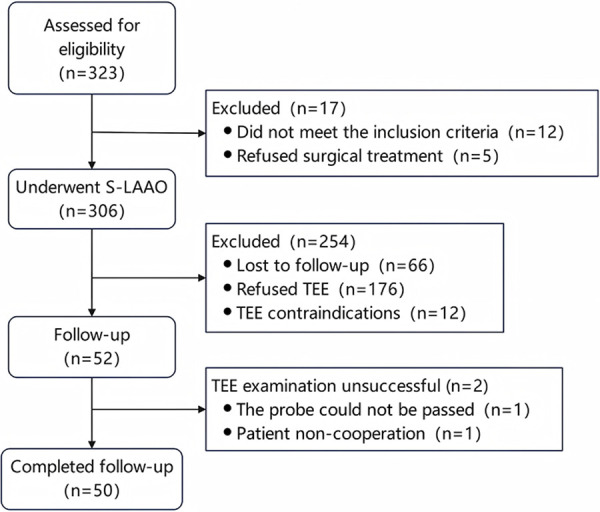
Flow diagram of patient selection and follow-up. TEE, transesophageal echocardiography; S-LAAO, surgical left atrial appendage occlusion.

The presence of thrombus in the LA or LAA was assessed using available preoperative echocardiographic data and was further confirmed by intraoperative TEE and direct surgical inspection before LAA closure. Thrombus location was recorded as LA thrombus, LAA thrombus, or thrombus involving both the LA and LAA. The patient screening and inclusion process is summarized in Figure 1.

### 2.3 Surgical procedures and operative data

All patients underwent valvular surgery under general anesthesia and cardiopulmonary bypass (CPB). Patients were placed in a supine position, and a median sternotomy was performed. CPB was established via cannulation of the aorta, and the superior and inferior vena cava. Intraoperative TEE was used to observe the functional status of each valve. The ascending aorta was clamped after reaching the target temperature, and circulation arrest was performed.

**Figure 2:**
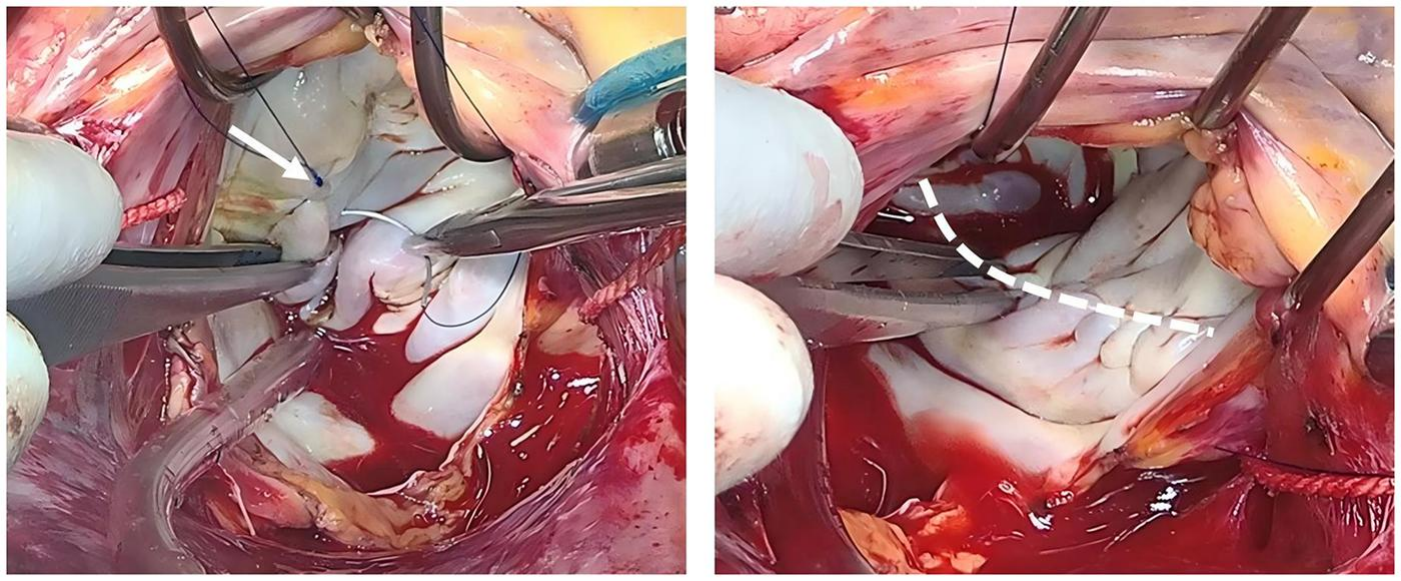
Intraoperative images of S-LAAO. The arrow indicates the starting point of endocardial suturing; the dashed line indicates the completed LAA closure line.

A bipolar radiofrequency clamp was utilized for linear ablation lines of both the left atrium (LA) and right atrium (RA), following the Cox-Maze IV ablation route, to obstruct the reentry ring and effectively treat AF (12). After the ablation procedure, an incision was made in the LA to expose its interior. A 4-0 polypropylene suture was used for continuous suturing along the junction of LA and LAA, from the upper to the lower margin. The single-layer simple running suture technique is employed, ensuring that the typical distance between the needle and neighboring needle does not exceed 2 mm. Strict control over strength, angle, and uniformity is imperative, while completing the procedure expeditiously (Figure 2).

Based on the intraoperative assessment of valve condition, repair or replacement of either aortic or mitral valve was performed post-ablation. In patients with severe coronary artery disease confirmed by preoperative coronary angiography, coronary artery bypass grafting (CABG) was conducted. After completing these operations, patients were gradually weaned off CPB. The remainder of the surgical procedure was performed as customary, including hemostasis, placement of temporary pacemaker leads for backup support, and closure of incisions.

### 2.4 TEE evaluation of LAA closure

Following intraoperative cardioversion, all patients underwent multiplane TEE to assess LAA closure from various angles (0 to 120°), conducted by an experienced echocardiologist. The evaluation included: (1) the assessment of continuous blood flow between LAA and LA using color Doppler; (2) the measurement of the maximum length of LAA post-closure. Criteria for closure failure were defined as a maximum stump length >1 cm or to-and-fro flow between LAA and LA, indicating early closure failure. While a maximum stump length >1 cm or to-and-fro flow were defined as closure failure according to established criteria, the clinical significance of smaller residual flows was also carefully examined in relation to thrombus formation.

### 2.5 Postoperative anticoagulation management

All patients received warfarin for postoperative anticoagulation management. Patients with mechanical valve replacement were prescribed lifelong warfarin anticoagulation. During the study period, warfarin therapy was managed according to the institutional low-intensity anticoagulation protocol for Chinese patients, with a target INR range of 1.8–2.5. This target range reflected local clinical practice and Chinese/Asian population evidence regarding the balance between thromboembolic prevention and bleeding risk. However, it is lower than contemporary North American and European guideline targets, particularly for patients with mechanical mitral valve replacement or additional thromboembolic risk factors. Therefore, INR values, time in therapeutic range (TTR), and anticoagulation status were further analyzed to contextualize thrombotic outcomes.

Patients who underwent bioprosthetic valve replacement or valvuloplasty received warfarin for 6 months according to the same institutional postoperative anticoagulation protocol, with a target INR range of 1.8–2.5. We acknowledge that this range should be interpreted as an institutional practice pattern rather than a universal guideline-based target for all valve-repair patients.

Patients who underwent CABG received aspirin (100 mg/day) in addition to their anticoagulation regimen. After completion of warfarin therapy, patients requiring antiplatelet therapy received aspirin and clopidogrel (75 mg/day) for 6 months, followed by long-term aspirin maintenance therapy.

### 2.6 Follow-up

Enrolled participants underwent follow-up examinations using both TTE and TEE. To assess the durability of LAA closure and distinguish between early technical failure and late recanalization, the timing of these examinations was carefully considered. All postoperative TEEs were performed cross-sectionally at the time of the final follow-up visit (mean 7.16 ± 1.73 years post-surgery). The same criteria were applied to assess successful or unsuccessful LAA closure. During TEE, the maximum length of any residual LAA stump was recorded, and the LA and residual LAA stump were carefully examined for thrombus. The presence of residual to-and-fro flow between the LA and LAA was assessed using color Doppler.

Rhythm status at follow-up was assessed using standard 12-lead electrocardiography at outpatient follow-up visits and by reviewing available inpatient and outpatient medical records. Holter monitoring was performed when clinically indicated. Recurrent AF was defined as documented AF on 12-lead electrocardiography, Holter monitoring, or medical records during follow-up. Antiarrhythmic drug use at the final follow-up was also recorded. Following postoperative assessments, all patients underwent regular INR testing as per protocol. Warfarin dosage adjustments were made under the guidance of anticoagulation specialists to maintain INR within the therapeutic range. The postoperative anticoagulation status of all patients was analyzed, including the duration of warfarin administration, the target INR range, and the occurrence of cerebral embolism. The time in therapeutic range (TTR) during oral warfarin administration was determined using the method of fraction of INRs in range in all patients. Embolic events were defined as clinical signs confirmed by computed tomography or angiography (neurological events).

### 2.7 Statistical analysis

Statistical analysis was conducted using IBM SPSS (Version 27.0) (IBM Corporation, Armonk, NY, USA). Descriptive statistics are presented as mean ± standard deviation (SD) or *n* (%), and categorical data as *n* (%). Paired comparisons of preoperative and postoperative continuous variables were performed using two-sided paired-samples t-tests. All statistical tests were two-sided, and a *P* value <0.05 was considered statistically significant.

## 3 Results

### 3.1 Operative data

A total of 50 patients were included in the final analysis, including 16 men and 34 women, with a mean age of 54.56 ± 7.71 years. The preoperative characteristics are summarized in Table 1. The mean preoperative LA diameter was 52.08 ± 10.60 mm, and the mean LVEF was 57.74 ± 7.28%. Persistent AF accounted for the largest proportion of AF type, occurring in 26 patients (52.0%).

**Table 1:** Preoperative patient characteristics.

Variable	*n* = 50
Age (years)	54.56 ± 7.71
Female, *n* (%)	34 (68%)
Body surface area (m^2^)	1.63 ± 0.18
BMI (kg/m^2^)	24.06 ± 3.66
LVEF (%)	57.74 ± 7.28
Diameter of the LA (mm)	52.08 ± 10.60
Serum creatinine (μmol/L)	76.32 ± 21.04
Diabetes, *n* (%)	5 (10%)
Hypertension, *n* (%)	8 (16%)
Cerebrovascular disease, *n* (%)	5 (10%)
Previous incidence of stroke, *n* (%)	1 (2%)
Chronic lung disease, *n* (%)	7 (14%)
NYHA class, *n* (%)	
Class 3	34 (68%)
Class 4	16 (32%)
Coronary heart disease, *n* (%)	11 (22%)
Previous catheter ablation, *n* (%)	0
Atrial fibrillation type, *n* (%)	
Paroxysmal	6 (12%)
Long-term persistence	18 (36%)
Persistent	26 (52%)

BMI, body mass index; LVEF, left ventricular ejection fraction; LA: left atrium; NYHA, New York Heart Association.

The mean CPB duration was 136.94 ± 38.43 min (range, 70.00–248.00 min), and the mean aortic cross-clamp time was 82.28 ± 26.97 min (range, 38.00–144.00 min). Valvular procedures included mitral valve procedures in 50 patients, aortic valve procedures in 14 patients, and tricuspid valve procedures in 35 patients. Mechanical valve replacement was performed in 28 patients, while biological valve replacement or valvuloplasty was performed in 22 patients. Other concomitant procedures included CABG in five patients and patent foramen ovale closure with patch repair in two patients (Table 2).

**Table 2:** Intraoperative patient data.

Variable	Result
Mitral valve treatment, *n* (%)	
Bio mitral	17 (34)
Mechanical mitral	28 (56)
Plasty mitral	5 (10)
Patient's classification, *n* (%)	
MVP	5 (10)
MVP, TVP	1 (2)
MVR	6 (12)
MVR, AVR	4 (8)
MVR, TVP	20 (40)
MVR, AVR, TVP	9 (18)
Concomitant CABG^a^	4 (8)
Concomitant CABG^b^	1 (2)
Perfusion time (min)	136.94 ± 38.43
Cross-clamp time (min)	82.28 ± 26.97
Prior thrombotic location, *n* (%)	
LAA	5 (10)
LA	4 (8)
Both	4 (8)

MVP, mitral valve plasty; TVP, tricuspid valve plasty; MVR, mitral valve replacement; AVR, aortic valve replacement; LAA, left atrial appendage; LA, left atrium; CABG, coronary artery bypass grafting.

a MVR, TVP.

b MVR, AVR, TVP.

Pre-existing LA and/or LAA thrombus was present in 13 patients (26.0%), including isolated LAA thrombus in 5 patients (10.0%), isolated LA thrombus in 4 patients (8.0%), and thrombus involving both the LA and LAA in 4 patients (8.0%). Intraoperative TEE confirmed no residual flow between the LAA and LA in any patient, and no LAA remnant was observed immediately after closure (Table 3).

**Table 3:** Left atrial appendage closure details.

Variable	Intraoperative TEE	Postoperative TEE
Mean follow-up (years)	–	7.16 ± 1.73
Residual stump ≤1 cm, *n* (%)	0	9 (18)
Residual stump >1 cm, *n* (%)	0	0
Residual flow, *n* (%)		
<3 mm	0	5 (10)
3–5 mm	0	0
>5 mm	0	0

TEE, transesophageal echocardiography; LA, left atrium.

### 3.2 Early clinical outcomes

There were no intraoperative or 30-day mortalities. Postoperative complications included atrioventricular block (*n* = 2 [4%]), peri-prosthetic leak (*n* = 2 [4%]), and re-exploration for bleeding (*n* = 1 [2%]). There were no incidences of renal insufficiency, cardiac tamponade, or stroke.

### 3.3 Medium-to-long-term TEE and TTE evaluation

**Figure 3:**
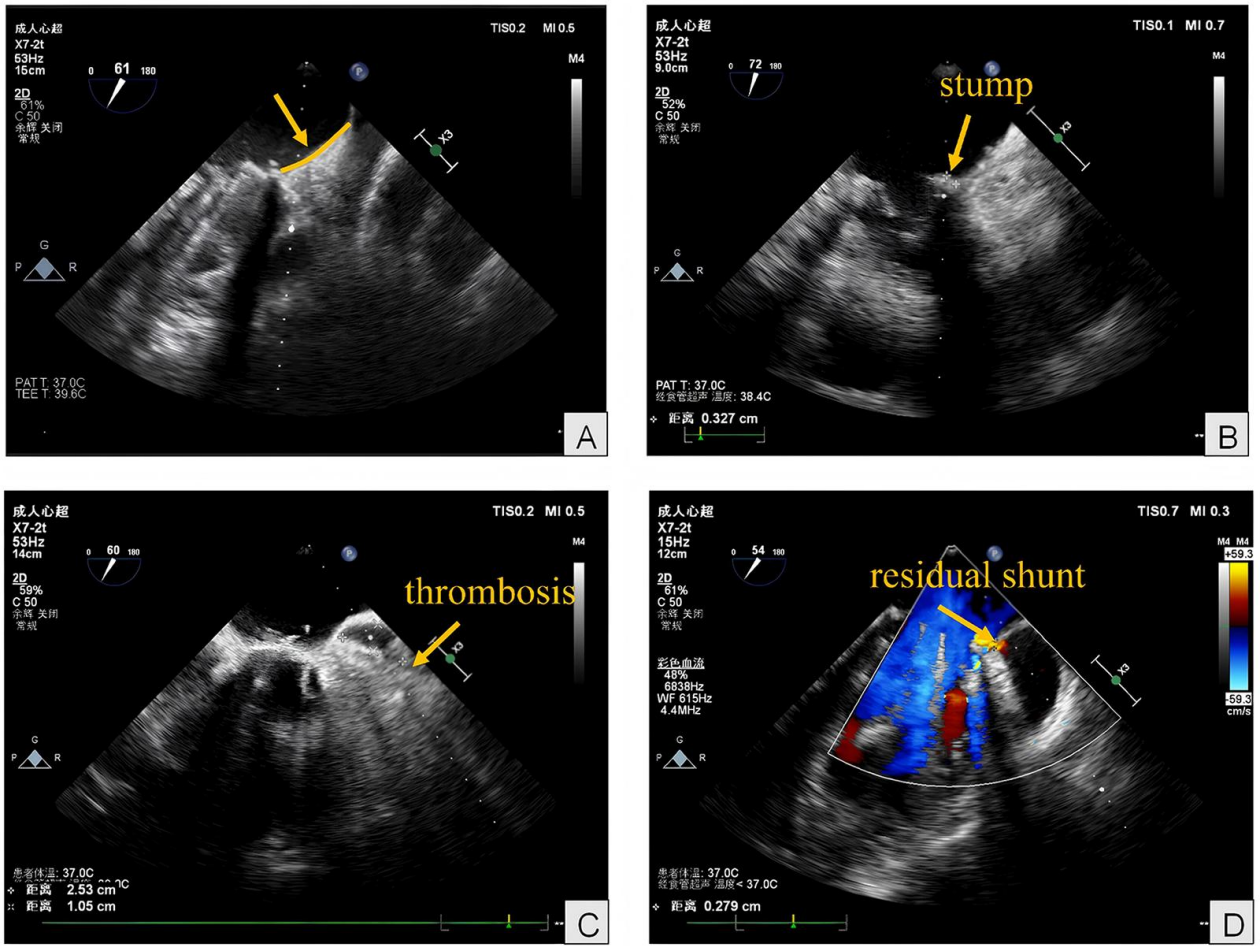
Intraoperative and postoperative evaluation of LAA closure. **(A)** Complete LAA closure without a residual stump or residual flow; **(B)** successful LAA closure with a residual stump <1 cm and no residual flow; **(C)** thrombus within the residual LAA stump; and **(D)** residual to-and-fro flow between the LA and residual LAA, with measurement of the maximum width of the residual flow channel.

Patients were followed up for a mean of 7.16 ± 1.73 years, with TTE and TEE used for postoperative assessment. The LA diameter decreased from 52.08 ± 10.60 mm preoperatively to 43.88 ± 6.89 mm at follow-up; this reduction remained statistically significant using a two-sided paired-samples t-test (mean reduction, 8.20 mm; 95% CI, 5.47–10.93; *P* < 0.001). During medium-to-long-term follow-up, TEE identified one case of bioprosthetic mitral valve degeneration. Residual LAA stumps were detected in 9 patients (18.0%), all with a maximum stump length <1 cm; no patient had a residual stump >1 cm. To-and-fro flow between the LA and LAA was observed in 5 patients (10.0%), with residual flow diameters ranging from 1 to 3 mm. Although none of the five patients with residual to-and-fro flow had a residual stump >1 cm, all met the study-defined criterion for closure failure because residual flow between the LA and LAA was present. Among these five patients, four (80.0%) developed thrombus within the residual LAA stump. Three of these four patients had undergone mechanical valve replacement, and one had undergone biological valve replacement (Table 3; Figure 3).

### 3.4 Follow-up data

At the final follow-up, rhythm status was available for all 50 patients. Sinus rhythm was maintained in 38 patients (76.0%), whereas recurrent AF was documented in 12 patients (24.0%). Among the 28 patients who underwent mechanical valve replacement, 23 (82.1%) were in sinus rhythm and 5 (17.9%) had recurrent AF. Among the 22 patients who received biological valve replacement or valvuloplasty, sinus rhythm was maintained in 15 (68.2%) and recurrent AF was documented in 7 (31.8%). At the final follow-up, 28 patients (56.0%) remained on oral anticoagulation, all of whom had undergone mechanical valve replacement. The remaining 22 patients (44.0%) had completed the planned 6-month course of warfarin after biological valve replacement or valvuloplasty and were not maintained on long-term oral anticoagulation. The mean TTR was 68.5% in patients with mechanical valve replacement and 70.8% in those with biological valve replacement or valvuloplasty. No bleeding complications were observed during warfarin administration. Antiarrhythmic drugs were used in 4 patients (8.0%) at the final follow-up, including amiodarone in 3 patients and propafenone in 1 patient.

No stroke events were reported among patients with successful LAA closure. Among the five patients with closure failure, none of the four patients with mechanical valve replacement experienced stroke. One patient with biological valve replacement experienced ischemic stroke 18 months postoperatively after completing the planned 6-month course of warfarin and was not receiving long-term oral anticoagulation at the time of stroke. The patient was in atrial fibrillation, and follow-up TEE showed a residual LAA stump <1 cm, residual to-and-fro flow of 1–2 mm, and thrombus within the residual LAA stump (Table 4).

**Table 4:** Postoperative follow-up findings.

	S-LAAO	Sinus rhythm	Recurrent AF	OAC at final follow-up	Stroke event
Mechanical VR (*n* = 28)	Success (*n* = 24)	20 (83.3%)	4 (16.7%)	24 (100%)	0
	Failure (*n* = 4)	3 (75%)	1 (25%)	4 (100%)	0
Biologic VR/Valvuloplasty (*n* = 22)	Success (*n* = 21)	14 (66.7%)	7 (33.3%)	0	0
	Failure (*n* = 1)^a^	1 (100%)	0	0	1 (100%)

S-LAAO, surgical left atrial appendage occlusion; AF, atrial fibrillation; VR, valve replacement.

At final follow-up, oral anticoagulation was continued in 28 patients (56.0%), all in the mechanical valve replacement group. Antiarrhythmic drugs were used in 4 patients (8.0%), including amiodarone in 3 and propafenone in 1.

a This patient had undergone biological valve replacement, completed the planned 6-month course of warfarin, and was not receiving long-term oral anticoagulation at the time of stroke. The patient was in atrial fibrillation, with a residual LAA stump <1 cm, small residual to-and-fro flow of 1–2 mm, and thrombus within the residual LAA stump on follow-up TEE.

## 4 Discussion

In patients with AF, intraoperative S-LAAO is pivotal in eliminating a thrombus-prone site. Available evidence suggests that successful intraoperative S-LAAO may reduce thromboembolic risk in selected patients with AF. Conversely, failure to close this structure may elevate the risk of strokes. Therefore, optimizing strategies to enhance the success rate of closure procedures becomes paramount.

Intraoperative and postoperative TEE revealed distinct reasons for the failure of various LAA closure techniques. Surgical excision, stapled excision, and AtriClip devices were more prone to failure due to a stump size >1 cm (13–15). Despite a stump size of <1 cm (16), there remains an increased risk of postoperative thrombus formation, leading to stroke events. The advantage of the endocardial suture technique lies in its minimal residual stump presence; however, suboptimal overall success rates have been reported, with persistent residual flow between LAA and LA being a common cause of failure. In our cohort, the medium-to-long-term success rate was 90.0% after a mean follow-up of 7.16 years, which compares favorably with several previously reported rates for endocardial suture closure. From our perspective, key factors contributing to enhanced success rates encompass shortening and ensuring consistency in continuous stitching. A single suture can be replaced with a double suture to reinforce isolation. For LAA openings with irregular shapes, suturing should ensure smoothness on the left atrial side so that uniform force is exerted at each suture line position during atrial contraction. Compared to other techniques for closing the LAA, the endocardial suture technique offers distinct advantages such as procedural simplicity, minimal bleeding, and cost-effectiveness. If durable closure can be achieved, this technique may remain a practical option in selected surgical settings.

Unlike Katz et al.'s findings (9), persistent blood flow signals were not observed between LA and LAA through TEE following restoration of heart rhythm. Such to-and-fro flow is hypothesized to manifest during the early postoperative period rather than intraoperatively. Among the five patients who experienced closure failure, residual flow port lengths ranging from 0.1 to 0.3 cm were observed, indicating leakage between adjacent sutures.

For patients with AF, long-term oral anticoagulation and LAA closure should be regarded as complementary rather than interchangeable strategies for stroke prevention. Current guidelines continue to recommend anticoagulation according to thromboembolic risk, even after rhythm control interventions or LAA closure, and percutaneous LAAO is mainly positioned as an alternative in selected patients with contraindications to long-term anticoagulation or high bleeding risk (5, 6, 17). Recent randomized evidence comparing LAA closure with medical therapy remains heterogeneous. In CLOSURE-AF, catheter-based LAA closure was not noninferior to physician-directed medical therapy in high-risk patients with AF (18). In contrast, CHAMPION-AF reported noninferiority of WATCHMAN FLX LAA closure compared with NOAC therapy in selected patients with nonvalvular AF, with lower nonprocedural bleeding but a numerically higher ischemic stroke signal (19). These trials involved percutaneous device-based closure and predominantly nonvalvular AF populations; therefore, their findings cannot be directly extrapolated to surgical endocardial suture closure in patients undergoing valve surgery. Nevertheless, these data reinforce that LAA closure should not be interpreted as a universal substitute for anticoagulation.

In the present cohort, anticoagulation strategies differed according to valve type. At the latest follow-up, sinus rhythm was documented in 76.0% of patients, with antiarrhythmic drug use in only 4 patients (8.0%), suggesting a favorable long-term rhythm status after concomitant Cox-Maze IV. Among patients who were not receiving long-term oral anticoagulation and had successful LAA closure (*n* = 21), 7 patients (33.3%) experienced recurrent AF; however, no stroke occurred during follow-up. The observed stroke proportion in this subgroup was 0.0%, but the exact 95% confidence interval was wide (0.0%–16.1%), indicating substantial uncertainty due to the limited sample size. This observation should be interpreted cautiously because the study was retrospective, the subgroup was small, and the number of stroke events was insufficient for formal risk estimation. Therefore, this finding should be regarded as hypothesis-generating and should not be interpreted as evidence that successful S-LAAO can routinely replace guideline-indicated anticoagulation.

Pre-existing LA/LAA thrombus should also be considered when interpreting postoperative thrombotic outcomes. In this cohort, 13 patients (26.0%) had pre-existing LA and/or LAA thrombus, indicating a substantial baseline thromboembolic substrate. Such patients may have had more advanced atrial remodeling, blood stasis, or systemic prothrombotic tendency, which could influence postoperative anticoagulation decisions and the subsequent risk of LAA stump thrombus or stroke. Because only five patients had residual to-and-fro flow, four developed LAA stump thrombus, and one experienced stroke, the number of events was insufficient for formal multivariable modelling. Therefore, the relationship between pre-existing LA/LAA thrombus and late LAA stump thrombus was assessed descriptively and should be interpreted cautiously. Larger studies with standardized preoperative TEE assessment are needed to determine whether pre-existing LA/LAA thrombus independently predicts closure failure, stump thrombosis, or thromboembolic events after surgical LAA closure.

Theoretically, residual flow between the LA and LAA may lead to blood stasis within the residual LAA stump. In the present study, thrombus formation within the residual LAA stump occurred in 4 of the 5 patients (80.0%) with closure failure. The single stroke event occurred in a patient with biological valve replacement who had completed the planned 6-month course of warfarin and was not maintained on long-term oral anticoagulation at the time of stroke. This patient had residual to-and-fro flow of 1–2 mm, thrombus within the residual LAA stump on follow-up TEE, and atrial fibrillation at the time of the event. These findings suggest that residual flow, recurrent AF, and absence of long-term anticoagulation may have jointly contributed to thrombus formation and embolization.

Although limited studies have directly examined the relationship between residual flow diameter after surgical endocardial suture LAA closure and thromboembolic events, percutaneous LAAO registries provide indirect evidence that small residual leaks may be clinically relevant. Alkhouli et al. reported that residual leaks <5 mm after percutaneous LAAO were associated with an increased risk of thromboembolic events (20). However, this comparison should be interpreted cautiously because percutaneous device-based LAAO and surgical endocardial suture closure have fundamentally different closure mechanisms and failure modes. Residual leaks after percutaneous LAAO usually reflect peri-device leak or incomplete device sealing, whereas residual flow after endocardial suture closure may result from suture-line leakage, tissue tearing, or late recanalization. Therefore, the NCDR LAAO registry data cannot be directly extrapolated to our surgical cohort and should be considered only as indirect supportive evidence. Accordingly, postoperative TEE should be used to evaluate both residual stump length and residual flow before anticoagulation decisions are considered. In patients with residual flow or unsuccessful closure, continuation of anticoagulation should be considered according to valve type, rhythm status, thromboembolic risk, bleeding risk, and imaging findings.

The formation of LAA stump thrombus in patients with mechanical valve replacement should also be interpreted in the context of anticoagulation intensity. The INR target range used in this cohort reflected our institutional low-intensity anticoagulation protocol for Chinese patients, consistent with the lower-intensity anticoagulation strategy commonly adopted in Chinese valve-surgery practice. However, this target range is lower than contemporary North American and European guideline recommendations, particularly for patients with mechanical mitral valve replacement, atrial fibrillation, or other thromboembolic risk factors. Therefore, low-intensity or subtherapeutic anticoagulation may have contributed to thrombus formation in some mechanical-valve patients. Nevertheless, all patients with LAA stump thrombus had residual to-and-fro flow between the LA and LAA, suggesting that incomplete functional closure may also have provided an important local thrombogenic substrate. Because of the small number of thrombotic events, the relative contributions of residual flow and anticoagulation intensity could not be disentangled.

An important finding of this study is that small residual to-and-fro flow may be clinically relevant even in the absence of a residual stump >1 cm. In this context, the observation that all residual flows were small (1–3 mm) yet frequently accompanied by thrombus highlights the potential clinical relevance of functional closure failure. These observations suggest that the functional status of LAA closure may be as important as morphological stump length.

The pathophysiological mechanism is plausible. Even a small residual communication may create a low-velocity, stagnant pocket within the residual LAA stump, thereby providing a nidus for thrombus formation. Therefore, the definition of successful surgical LAA closure should incorporate both morphological and functional criteria. Relying solely on a stump length <1 cm may be insufficient. The presence of any detectable residual flow between the LA and LAA, regardless of stump length, should be regarded as a high-risk imaging feature for thrombus formation.

Accordingly, postoperative TEE should not only measure residual stump length but also carefully evaluate residual flow using color Doppler. In patients with residual flow, anticoagulation discontinuation should be approached cautiously, and continued anticoagulation should be considered according to valve type, rhythm status, thromboembolic risk, bleeding risk, and imaging findings.

## 5 Limitations

Our study has several limitations. First, the relatively low INR target range represents an important limitation. Although low-intensity warfarin anticoagulation has been adopted in Chinese valve-surgery practice and is supported by Chinese/Asian population data, the INR target range used in this study was lower than contemporary North American and European guideline recommendations, especially for patients with mechanical mitral valve replacement, atrial fibrillation, or other thromboembolic risk factors. Therefore, low-intensity or subtherapeutic anticoagulation may have contributed to LAA stump thrombus formation in some patients, particularly those with mechanical valve replacement. Our findings should not be interpreted as supporting a lower INR target, and anticoagulation management should remain individualized and guideline-informed.

Second, more than half of the eligible patients declined follow-up TEE and were therefore excluded from the final analysis, which may have introduced selection bias. Future studies should consider strategies to improve compliance with follow-up imaging or use cardiac computed tomography angiography as an alternative imaging modality in patients who decline TEE.

Third, the relatively small follow-up sample size limits the statistical power and generalizability of our findings, particularly for thrombotic and stroke outcomes. In particular, the subgroup of patients with successful LAA closure who were not maintained on long-term oral anticoagulation was small; although no stroke occurred in these 21 patients, the exact 95% confidence interval was wide (0.0%–16.1%), indicating limited precision.

Fourth, rhythm status was assessed using clinically available ECG-based data rather than continuous rhythm monitoring in all patients. Therefore, asymptomatic or intermittent AF recurrence may have been underestimated, which may affect the interpretation of the relationship among rhythm status, LAA closure, anticoagulation discontinuation, and stroke risk.

Finally, this study included only patients with valvular heart disease undergoing concomitant valve surgery, Cox-Maze IV ablation, and endocardial LAA closure. Therefore, caution is warranted when extrapolating these findings to patients with non-valvular AF, isolated LAA closure, or other cardiac conditions.

## 6 Conclusion

Endocardial suture closure of the LAA during valve surgery and Cox-Maze IV ablation demonstrated a high medium-to-long-term anatomical success rate in this selected retrospective cohort. However, small residual to-and-fro flow was associated with LAA stump thrombus, even when the residual stump was <1 cm. Therefore, postoperative TEE should evaluate both residual stump length and residual flow before anticoagulation decisions are considered. Given the limited sample size and retrospective design, the absence of stroke among patients with successful LAA closure who were not maintained on long-term oral anticoagulation should be regarded as hypothesis-generating rather than practice-changing. Anticoagulation management should remain individualized and guideline-informed.

## Data Availability

The original contributions presented in the study are included in the article. Further inquiries can be directed to the corresponding authors.

## References

[1] Odutayo A Wong CX Hsiao AJ Hopewell S Altman DG Emdin CA. Atrial fibrillation and risks of cardiovascular disease, renal disease, and death: systematic review and meta-analysis. Br Med J. (2016) 354:i4482. 10.1136/bmj.i4482 27599725

[2] Blackshear JL Odell JA. Appendage obliteration to reduce stroke in cardiac surgical patients with atrial fibrillation. Ann Thorac Surg. (1996) 61(2):755–9. 10.1016/0003-4975(95)00887-x 8572814

[3] Park-Hansen J Holme SJV Irmukhamedov A Carranza CL Greve AM Al-Farra G. Adding left atrial appendage closure to open heart surgery provides protection from ischemic brain injury six years after surgery independently of atrial fibrillation history: the LAACS randomized study. J Cardiothorac Surg. (2018) 13(1):53. 10.1186/s13019-018-0740-7 29792215 PMC5967101

[4] Aryana A Singh SK Singh SM O'Neill PG Bowers MR Allen SL. Association between incomplete surgical ligation of left atrial appendage and stroke and systemic embolization. Heart Rhythm. (2015) 12(7):1431–7. 10.1016/j.hrthm.2015.03.028 25998141

[5] Whitlock RP Belley-Cote EP Paparella D Healey JS Brady K Sharma M. Left atrial appendage occlusion during cardiac surgery to prevent stroke. N Engl J Med. (2021) 384(22):2081–91. 10.1056/NEJMoa2101897 33999547

[6] Joglar JA Chung MK Armbruster AL Benjamin EJ Chyou JY Cronin EM. 2023 ACC/AHA/ACCP/HRS guideline for the diagnosis and management of atrial fibrillation: a report of the American College of Cardiology/American Heart Association Joint Committee on clinical practice guidelines. Circulation. (2024) 149(1):e1–e156. 10.1161/cir.0000000000001193 38033089 PMC11095842

[7] Kabra R Gopinathannair R Lakkireddy D. Left atrial appendage occlusion during cardiac surgery: a 75-year-old journey. J Am Heart Assoc. (2023) 12(10):e030127. 10.1161/jaha.123.030127 37183870 PMC10227286

[8] Squiers JJ Edgerton JR. Surgical closure of the left atrial appendage: the past, the present, the future. J Atr Fibrillation. (2018) 10(5):1642. 10.4022/jafib.1642 29988257 PMC6006968

[9] Katz ES Tsiamtsiouris T Applebaum RM Schwartzbard A Tunick PA Kronzon I. Surgical left atrial appendage ligation is frequently incomplete: a transesophageal echocardiograhic study. J Am Coll Cardiol. (2000) 36(2):468–71. 10.1016/s0735-1097(00)00765-8 10933359

[10] García-Fernández MA Pérez-David E Quiles J Peralta J García-Rojas I Bermejo J. Role of left atrial appendage obliteration in stroke reduction in patients with mitral valve prosthesis: a transesophageal echocardiographic study. J Am Coll Cardiol. (2003) 42(7):1253–8. 10.1016/s0735-1097(03)00954-9 14522491

[11] Kanderian AS Gillinov AM Pettersson GB Blackstone E Klein AL. Success of surgical left atrial appendage closure: assessment by transesophageal echocardiography. J Am Coll Cardiol. (2008) 52(11):924–9. 10.1016/j.jacc.2008.03.067 18772063

[12] Damiano RJ Jr Schwartz FH Bailey MS Maniar HS Munfakh NA Moon MR. The cox maze IV procedure: predictors of late recurrence. J Thorac Cardiovasc Surg. (2011) 141(1):113–21. 10.1016/j.jtcvs.2010.08.067 21168019 PMC3035158

[13] Ahmed A Pothineni NVK Singh V Bawa D Darden D Kabra R. Long-term imaging and clinical outcomes of surgical left atrial appendage occlusion with AtriClip. Am J Cardiol. (2023) 201:193–9. 10.1016/j.amjcard.2023.06.026 37385174

[14] Kang Y Hwang HY Joo S Park JH Kim JS Sohn SH. Left atrial appendage elimination techniques: stapled excision versus internal suture obliteration. J Thorac Dis. (2021) 13(11):6252–60. 10.21037/jtd-21-1138 34992805 PMC8662504

[15] Lee R Vassallo P Kruse J Malaisrie SC Rigolin V Andrei AC. A randomized, prospective pilot comparison of 3 atrial appendage elimination techniques: internal ligation, stapled excision, and surgical excision. J Thorac Cardiovasc Surg. (2016) 152(4):1075–80. 10.1016/j.jtcvs.2016.06.009 27422360

[16] Hui DS Alderson LJ Lee R. Left atrial appendage thrombus after successful surgical exclusion on anticoagulation: a need for closer postintervention monitoring. Ann Thorac Surg. (2014) 98(4):1478. 10.1016/j.athoracsur.2014.06.075 25282222

[17] Saw J Holmes DR Cavalcante JL Freeman JV Goldsweig AM Kavinsky CJ. SCAI/HRS expert consensus statement on transcatheter left atrial appendage closure. Heart Rhythm. (2023) 20:e1–e16. 10.1016/j.hrthm.2023.01.007 36990925

[18] Landmesser U Skurk C Kirchhof P Lewalter T Hartung J Rroku A. Left atrial appendage closure or medical therapy in atrial fibrillation. N Engl J Med. (2026) 394(13):1270–80. 10.1056/NEJMoa2513310 41849741

[19] Doshi SK Kar S Nair DG Waggoner T Agarwal H Moussavian M. Left atrial appendage closure or anticoagulation for atrial fibrillation. N Engl J Med. (2026) 394(21):2083–94. 10.1056/NEJMoa2517213 41910347

[20] Alkhouli M Du C Killu A Simard T Noseworthy PA Friedman PA. Clinical impact of residual leaks following left atrial appendage occlusion: insights from the NCDR LAAO registry. JACC Clin Electrophysiol. (2022) 8(6):766–78. 10.1016/j.jacep.2022.03.001 35387751 PMC9233062

